# Retirement as a predictor of physical functioning trajectories among older businessmen

**DOI:** 10.1186/s12877-022-03001-x

**Published:** 2022-04-04

**Authors:** Markus J. Haapanen, Timo E. Strandberg, Timo Törmäkangas, Monika E. von Bonsdorff, Arto Y. Strandberg, Mikaela B. von Bonsdorff

**Affiliations:** 1 Department of General Practice and Primary Health Care, University of Helsinki and Helsinki University Hospital, PO Box 20, FI-00014 Helsinki, Finland; 2 Folkhälsan Research Center, Helsinki, Finland; 3 Department of Medical Epidemiology and Biostatistics, Karolinska Institutet, Stockholm, Sweden; 4 Department of Medicine, Helsinki University Hospital, University of Helsinki, Helsinki, Finland; 5 Center for Life Course Health Research, University of Oulu, Oulu, Finland; 6 Gerontology Research Center and Faculty of Sport and Health Sciences, University of Jyväskylä, Jyväskylä, Finland; 7 School of Business, University of Jyväskylä, Jyväskylä, Finland

**Keywords:** Age at retirement, Type of pension, Physical functioning

## Abstract

**Background:**

Associations between retirement characteristics and consequent physical functioning (PF) are poorly understood, particularly in higher socioeconomic groups, where postponing retirement has had both positive and negative implications for PF.

**Methods:**

Multiple assessments of PF, the first of which at the mean age of 73.3 years, were performed on 1709 men who were retired business executives and managers, using the RAND-36/SF-36 instrument, between 2000 and 2010. Questionnaire data on retirement age and type of pension was gathered in 2000. Five distinct PF trajectories were created using latent growth mixture modelling. Mortality- and covariate-adjusted multinomial regression models were used to estimate multinomial Odds Ratios (mOR) on the association between retirement characteristics and PF trajectories.

**Results:**

A one-year increase in retirement age was associated with decreased likelihood of being classified in the ‘consistently low’ (fully adjusted mOR = 0.82; 95%CI = 0.70, 0.97; *P* = 0.007), ‘intermediate and declining’ (mOR = 0.89; 95%CI = 0.83, 0.96; *P* = 0.002), and ‘high and declining’ (mOR = 0.92; 95%CI = 0.87, 0.98; *P* = 0.006) trajectories, relative to the ‘intact’ PF trajectory. Compared to old age pensioners, disability pensioners were more likely to be classified in the ‘consistently low’ (mOR = 23.77; 95% CI 2.13, 265.04; *P* = 0.010), ‘intermediate and declining’ (mOR = 8.24; 95%CI = 2.58, 26.35; *P* < 0.001), and ‘high and declining’ (mOR = 2.71; 95%CI = 1.17, 6.28; *P* = 0.020) PF trajectories, relative to the ‘intact’ PF trajectory.

**Conclusions:**

Among executives and managers, older age at retirement was associated with better trajectories of PF in old age. Compared to old age pensioners, those transitioning into disability and early old age pensions were at risk of having consistently lower PF in old age.

**Supplementary Information:**

The online version contains supplementary material available at 10.1186/s12877-022-03001-x.

## Key Points

- Transitioning into retirement has been associated with changes in consequent physical functioning (PF) in different occupational and socioeconomic groups.
- Retirement at and older age associated with better PF trajectories among former executives and managers in old age. Compared to old age pensioners, disability- and early old age pensioners were at risk of having consistently lower PF in old age.
- Retiring at an older age didn’t appear to be harmful for PF among men in higher occupational groups

## Introduction

Physical functioning (PF) is essential for maintaining independence and determining the quality of life in old age. Associations between poor PF and increased risk of nursing home admissions, disability, geriatric syndromes, and mortality [1–3], highlight the need to focus on functioning among older adults. Physical functioning has been observed to decline faster among the retired than those still in full-time work [4]. A key life event, retirement may allow readjustment to how physical activity is perceived psychologically [5] and reduce lifestyle risk behaviours including physical inactivity [6], enabling more sustained levels of PF.

The existing literature on the association between retirement and PF shows that the relationships may vary by occupational and socioeconomic factors [7–10]. Whereas younger age at retirement may even be beneficial for the recovery of PF of individuals in poor health [9, 10], in another study the PF of disability pensioners continued to decline during retirement [8]. Results among individuals in higher occupational groups, and among those in better health, have likewise proven inconclusive. While some suggest associations to be qualitatively similar across socioeconomic groups [7], some report better and more stable PF among higher social class representatives [10], whereas others found negative consequences of retirement on PF among healthy and wealthy participants [9]. To conceive retirement policies that promote functioning, more research is warranted in areas characterized with varying results, e.g. among higher socioeconomic and occupational groups. Our aim was to gain better insight into the association between retirement and post-retirement PF trajectories in higher occupational groups. We followed up 10-year PF trajectories of 1709 retired executives and managers into old age. Moreover, regarding types of pension, we are not aware of a previous study to assess relationships between unemployment pensions, receipt of individual retirement packages, and PF. Our hypothesis was that 1) older age at retirement would associate with more stable PF trajectories in old age and 2) old age pensioners would maintain their PF better than those receiving early old age, individual retirement package, unemployment, and disability pensions. The rationale behind our hypotheses was that sustained working ability, as indicated in older age at retirement and receipt of old age pensions, would also act as a broader indicator of health status and therefore enable a more stable PF.

## Methods

### Study population

The Helsinki Businessmen Study (HBS) [11, 12] includes 3310 men born during 1919–1934. The men had been business executives and managers during their careers and participated in voluntary health check-ups at the Finnish Institute of Occupational Health between 1964 and 1973. Survivors (*n* = 2287) received mailed questionnaires (including the RAND-36/SF-36 questionnaire) in data collection waves organized in 2000, 2003, 2007, and 2010 (response rates were 81.5, 66.3, 65.1, and 67.8%, respectively). Self-reported data on age at retirement and type of pension was gathered in the year 2000. The analytical sample consisted of 1709 men with complete retirement and PF trajectory data. The research protocol of the follow-up study of HBS has been approved by the Ethics Committee of the Department of Medicine, University of Helsinki. The study adheres with the principles stated in the Declaration of Helsinki. Written informed consent was obtained from all study participants before initiating any study procedures.

### Physical functioning

Physical functioning was assessed at baseline in the year 2000 (*n* = 1698, mean age 73.3 years; SD 4.1 years) using the 10 items in the Physical functioning domain of the validated RAND-36 Health Survey [13, 14], v.1.0 (identical with Short Form SF-36) [15]. Questions on how the participants’ health limited their daily activities, e.g. walking 2.5 km or 100 m, or climbing one or more flights of stairs, were embedded in the survey. Response alternatives ‘not limited at all’, ‘limited a little’, and ‘limited a lot’, were given numeral values; 100, 50, and 0, respectively. The sum value was divided by 10 to obtain a score (range = 0–100), in which a higher score indicated better physical functioning. A minimum of 7 of the 10 physical functioning items were required from each data collection wave for the score to be calculated and divided by the respective number of answered questions.

**Fig. 1:**
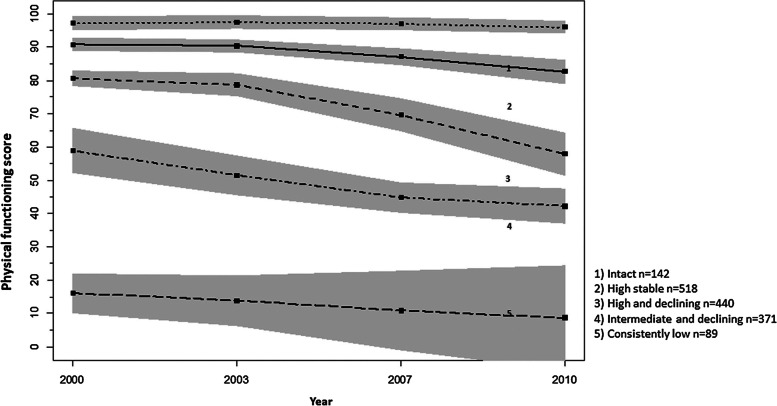
Identified physical functioning trajectories over the 10-year follow-up from 2000 to 2010. Reproduced with permission from [15]

We used latent growth mixture models (LGMM) with full information maximum likelihood to capture latent groups in our data that had similar PF trajectories but that were distinct over the follow-up period. Their creation process has been described in detail previously [15]. Briefly, latent groups had each their own growth parameters, including intercept and slope, and they were estimated for quadratic and cubic shapes to find all potential differences in PF. Model fit indices showed the best fit for a five-class solution, as shown in model fit statistics presented in Supplementary Table 1 Grouping was based on the probability of the membership calculated for each participant’s individual trajectory. The five PF trajectories were named ‘intact’, ‘high stable’, ‘high and declining’, ‘intermediate and declining’ and ‘consistently low’ (Fig. 1). Individual observations belonging to each PF trajectory are presented in Supplementary Fig. 1.

### Age at retirement and type of pension benefit

Participants answered questions on retirement, e.g. exact retirement age in years and the type of pension benefit, in the mailed questionnaire in the year 2000. By that time, 95.9% (*n* = 1784) of the men reported that they had retired. Information on occupation or the age which was considered ‘statutory retirement age’ in these sectors/occupations, was not known. We identified 6 types of pensions: old age pension (*n* = 632; 37.0%), early old age pension (*n* = 359; 21.0%), individual deal for a pension package (*n* = 323; 18.9%), part-time pension (*n* = 16; 0.9%), unemployment pension (*n* = 129; 7.5%) and disability pension (*n* = 250; 14.6%). The category ‘part-time pension’ had few observations and was merged with the category ‘early old age pension’, as these groups were otherwise similar regarding background information.

For this Finnish population, the lower age limit for old age pension benefits was higher for private sector (65 years) than public sector (63 years) workers. Employees may also have had old age pension schemes where these age limits varied, mostly between age 60 and 65 years. Early old age pensions and part-time pensions offer flexibility and have a lower age limit; 60 years for private and 58 years for public sector workers. Unemployment pensions were granted following 500 days of unemployment allowance, at minimum age 55–60 years, to Finnish citizens born before 1950. Disability pensions require a physician-diagnosed disability that continually reduces work capacity for over 300 days. Normal disability pensions can be granted to Finns aged 16 years or older with significant disability. Relaxed disability pensions require a minimum age of 55 years, a long work career and a medical condition that causes at least partial disability [16].

### Covariates

Questionnaire data on covariates were from the year 2000. Self-reported height in metres and weight in kilograms were used to calculate body mass index (BMI), expressed as kg/m^2^. Smoking status was coded into current smokers, ex-smokers, and never smokers. Weekly consumption of grams of pure alcohol was used to divide participants into abstainers, light, moderate, or high consumers (cut-offs 98 and 196 g/week) [17]. Leisure-time physical activity (LTPA) was assessed with the questions “Do you exercise regularly weekly?”, “If yes, how many hours per week?” and “How many times a week do you have exercise leading to sweating and breathlessness?”. We used weekly hours of LTPA to categorize participants as low, moderate and highly active (cut-offs at 2 and 6 h of LTPA/week) [18]. The participants provided information on physician-diagnosed chronic illness, including cardiovascular disease and diabetes, in the questionnaire. Mortality data from the beginning of the year 2000 until 31st December 2010 were retrieved from the Finnish Population Information System, which is a national register comprising all Finnish citizens.

### Statistical methods

The data are presented as means and standard deviations (SD) for continuous variables, and as percentages for categorical variables. Among continuous variables differences between group central tendency statistics (i.e. means and mean ranks) were tested using analyses of variance and Kruskall-Wallis test for normally and non-normally distributed variables, respectively. Mortality- and covariate-adjusted multinomial regression models were used to estimate multinomial Odds Ratios (mOR) and 95% confidence intervals, representing adjusted relationships for trajectory group membership probabilities, with *P* values for the associations. The models were adjusted for age, alcohol use, smoking, LTPA, BMI, diabetes, cardiovascular disease and for the latent effect of excess mortality risk between 2000 and 2010. Proportionality was supported by Kaplan-Meier curves not crossing significantly. We tested proportionality using scaled Schoenfeld residuals where nonsignificant *P* values support proportionality. Proportionality was supported for all covariates with an effect in the mortality part of the model, as well as with the global estimate of proportionality. The analyses were two-tailed, and significance was set at 0.05. Mixture modelling and mortality-adjusting multinomial regression modelling were conducted in Mplus (v 7.0). All other analyses were carried out using statistical software SPSS (IBM SPSS Statistics, version 25.0 released 2017; IBM Corp, Armonk, NY).

## Results

The 1709 men had retired at the mean age of 61.2 years (SD = 4.4, range = 27–76 years) and most received old age pensions (37.0%). The mean RAND-36 PF scale score was 75.5 (SD = 23.2, range = 0–100 points) at mean age 73.3 years (SD = 4.0, range = 66–81 years) in the year 2000. More than one in three died (*n* = 601, 35.2%) during the observation period between 2000 and 2010. (data not shown).

The five identified PF trajectories were called as follows: ‘intact’ (8.6%), ‘high and stable’ (32.2%), ‘high and declining’ (29.5%), ‘intermediate and declining’ (23.8%) and ‘consistently low’ (5.9%). Table 1 summarizes the participants’ characteristics according to the five PF trajectories. The difference in age, smoking, alcohol consumption, LTPA, cardiovascular disease and diabetes across the PF trajectory groups was statistically significant and generally suggested that men in the ‘intact’ PF category were healthier and younger and that the participants’ health status worsened with declining PF trajectories (*p*-values≤0.007, Table 1). Mortality during the 10-year observation period generally increased with worsening PF trajectory groups, being 15.0% in the category ‘intact’ and 83.2% among men with ‘consistently low’ PF (*p* < 0.001, Table 1).

**Table 1:** Characteristics of the men according to physical functioning trajectories during retirement (proportion and percent unless stated otherwise)

	Physical functioning trajectories					*P*-value^a^
	Intact *n* = 147	High stable *n* = 551	High and declining *n* = 504	Intermediate and declining *n* = 406	Consistently low *n* = 101	
**Old-age characteristics, assessed in the year 2000**						
Age, average years (SD)	71.0 (3.5)	72.4 (3.8)	73.7 (3.9)	74.3 (4.1)	74.8 (4.0)	< 0.001
Smoking status, n (%)						< 0.001
Never smoker	73 (50.0)	227 (41.2)	170 (33.7)	134 (33.0)	26 (25.7)	
Ex-smoker	66 (45.2)	282 (51.2)	292 (57.9)	223 (54.9)	63 (62.4)	
Current smoker	7 (4.8)	42 (7.6)	42 (8.3)	49 (12.1)	12 (11.9)	
Alcohol consumption, n (%)						0.007
None	54 (36.7)	167 (30.3)	181 (35.9)	159 (39.2)	49 (48.5)	
Light	57 (38.8)	185 (33.6)	164 (32.5)	117 (28.8)	27 (26.7)	
Moderate	22 (15.0)	108 (19.6)	92 (18.3)	62 (15.3)	10 (9.9)	
High	14 (9.5)	91 (16.5)	67 (13.3)	68 (16.7)	15 (14.9)	
LTPA, n (%)						< 0.001
Low	2 (1.5)	16 (3.5)	18 (4.4)	13 (4.9)	9 (25.0)	
Moderate	72 (54.5)	247 (53.5)	249 (61.0)	174 (65.9)	22 (61.1)	
High	58 (43.9)	199 (43.1)	141 (34.6)	77 (29.2)	5 (13.9)	
Diabetes, n (%)	5 (3.4)	31 (5.6)	49 (9.7)	57 (14.0)	35 (34.7)	< 0.001
CVD, n (%)	47 (32.0)	299 (54.3)	340 (67.5)	298 (73.4)	87 (86.1)	< 0.001
**Retirement characteristics**						
Age at retirement, average years (SD)	61.5 (4.6)	61.5 (4.4)	61.1 (4.0)	60.9 (4.5)	59.9 (5.4)	0.020
Type of pension benefit						< 0.001
Old age	59 (40.1)	211 (38.3)	186 (36.9)	142 (35.0)	34 (33.7)	
Early old age	23 (15.6)	122 (22.1)	122 (24.2)	91 (22.4)	17 (16.8)	
Individual retirement package	27 (18.4)	130 (23.6)	88 (17.5)	67 (16.5)	11 (10.9)	
Unemployment	27 (18.4)	38 (6.9)	39 (7.7)	20 (4.9)	5 (5.0)	
Disability	11 (7.5)	50 (9.1)	69 (13.7)	86 (21.2)	34 (33.7)	
**Mortality, assessed between years 2000–2010**						
Died, n (%)	22 (15.0)	117 (21.2)	156 (31.0)	222 (54.7)	84 (83.2)	< 0.001

*Note*. *SD* standard deviation, *LTPA* leisure time physical activity

^a^P for difference between physical functioning trajectories

### Age at retirement and physical functioning trajectories

**Fig. 2:**
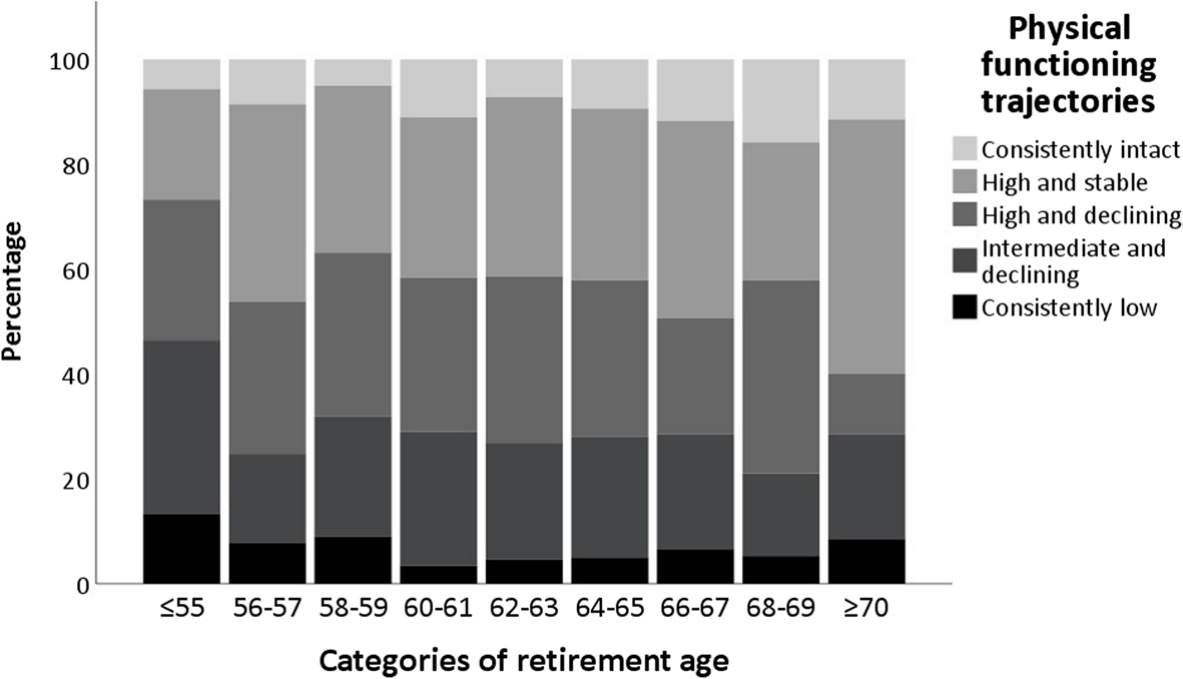
Distribution of physical functioning trajectories according to categories of retirement age

Mean age at retirement decreased across worsening PF trajectory groups; it was 61.5 years for ‘intact’, 61.5 years for ‘high and stable’, 61.1 years for ‘high and declining’, 60.9 years for ‘intermediate and declining’, and 59.9 years for ‘consistently low’ (*p* = 0.020, Table 1). Figure 2 illustrates the proportionate distribution of PF categories across 2-year groups of retirement age.

Table 2 presents associations between age at retirement and PF trajectories for the mortality- and covariate-adjusted multinomial regression models. A one-year increase in retirement age was associated with decreased likelihood of being classified in the ‘consistently low’ (fully adjusted mOR = 0.82; 95% CI 0.70, 0.97; *P* = 0.007), ‘intermediate and declining’ (fully adjusted mOR = 0.89; 95% CI 0.83, 0.96; *P* = 0.002), and ‘high and declining’ (fully adjusted mOR = 0.92; 95% CI 0.87, 0.98; *P* = 0.006) trajectories, relative to the ‘intact’ PF trajectory. The association regarding the ‘high and stable’ trajectory was similar but not statistically significant (fully adjusted mOR = 0.97; 95% CI 0.92, 1.02; *P* = 0.263).

**Table 2:** Multinomial Odds Ratios (mOR), 95% confidence intervals, and *P* values for path coefficients of models for age at and type of pension predicting physical functioning trajectories in old age in the Helsinki Businessmen Study

	High stable vs intact			High and declining vs intact			Intermediate and declining vs intact			Consistently low vs intact		
	mOR	95% CI	*p*	mOR	95% CI	*p*	mOR	95% CI	*p*	mOR	95% CI	*p*
**Retirement age**^**a**^												
In years	0.97	0.92, 1.02	0.263	0.92	0.87, 0.98	0.006	0.89	0.83, 0.96	0.002	0.82	0.70, 0.97	0.007
**Type of pension**^**a**^												
Old age	Ref.			Ref.			Ref.			Ref.		
Early old age	1.54	0.85, 2.77	0.152	2.13	1.13, 4.02	0.019	2.83	1.23, 6.53	0.015	1.09	0.15, 7.80	0.933
Individual retirement package	1.70	0.94, 3.07	0.077	1.48	0.78, 2.83	0.704	1.57	0.68, 3.67	0.294	1.16	0.17, 8.07	0.149
Unemployment	0.55	0.28, 1.10	0.095	0.86	0.40, 1.85	0.232	0.37	0.10, 1.34	0.130	0.16	0.01, 7.29	0.348
Disability	1.54	0.70, 3.39	0.286	2.71	1.17, 6.28	0.020	8.24	2.58, 26.35	< 0.001	23.77	2.13, 265.04	0.010

*Note*. *mOR* multinomial odds ratio, *CI* confidence interval

^a^Adjusted for age in 2000, alcohol use, smoking, LTPA, BMI, diabetes, cardiovascular disease and mortality risk between the years 2000 and 2010

### Type of pension benefit and physical functioning trajectories

**Fig. 3:**
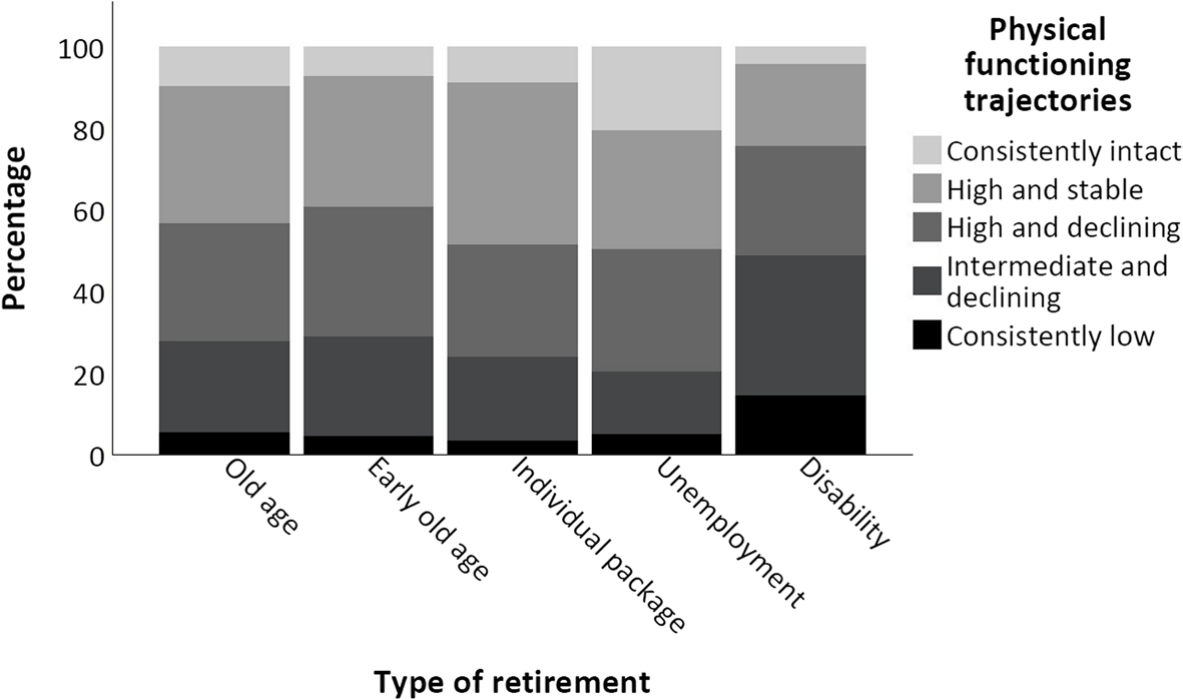
Distribution of physical functioning trajectories according to categories of pension benefit

The difference in the proportions of types of pension benefit across PF trajectory groups was statistically significant and generally showed that the proportions of old age pensioners decreased and those of disability pensioners increased with worsening PF trajectory groups (*p* < 0.001, Table 1). The distribution of early old age pensioners roughly followed an inverted U-shaped distribution, being highest (24.2%) in the ‘high and declining’ PF trajectory and lowest in the categories ‘intact’ (15.6%) and ‘consistently low’ (16.8%). Receipt of an individual retirement package was most common in the ‘high and stable’ PF trajectory, and least common among those classified with ‘consistently low’ PF. The prevalence of unemployment pensioners was more than 2-fold higher (18.4%) in the trajectory ‘intact’ than in any other trajectory. Figure 3 presents the proportionate distributions of PF trajectories according to the type of pension benefit.

Table 2 presents the associations between the type of pension benefit and PF trajectories, in multinomial regression models accounting for excess mortality risk. Compared to old-age pensioners, disability pensioners were more likely to be classified in the ‘consistently low’ (fully adjusted mOR = 23.77; 95% CI 2.13, 265.04; *P* = 0.010), ‘intermediate and declining’ (fully adjusted mOR = 8.24; 95% CI 2.58, 26.35; *P* < 0.001), and ‘high and declining’ (fully adjusted mOR = 2.71; 95% CI 1.17, 6.28; *P* = 0.020) PF trajectories, relative to the ‘intact’ PF trajectory. The coefficient for disability pensioners concerning the ‘high and stable’ PF trajectory was also greater than one but non-significant. In contrast, compared to old age pensioners, those receiving unemployment pensions were at increased probability of maintaining their PF, as indicated by smaller though non-significant point estimates (fully adjusted mOR’s from 0.16 to 0.86) of membership probability across the PF trajectories, relative to the ‘intact’ PF trajectory. Membership probability across the PF trajectories did not differ statistically significantly among men receiving individual retirement packages, comparing with old age pensioners. Compared to old age pensioners, those receiving early old age pensions were more likely to be classified in the ‘intermediate and declining’ (fully adjusted mOR = 2.83; 95% CI 1.23, 6.53 *P* = 0.015) and ‘high and declining’ (fully adjusted mOR = 2.13; 95% CI 1.13, 4.02; *P* = 0.019) PF trajectories, relative to the ‘intact’ PF trajectory.

## Discussion

Our results show that both age at retirement as well as the type of pension are linked with subsequent development of physical functioning across old age. We identified five distinct PF trajectories over a 10-year period in this longitudinal cohort study consisting of former business executives and managers, who were first assessed for RAND-36/SF-36 PF at a mean age of 73.3 years and then followed up for 10 years. Older age at retirement was associated with a higher probability to belong in a group with a favourable average PF trajectory in old age. Compared to old age pensioners, those receiving disability pension were more likely to belong in a group with declining or low average PF trajectory in old age. Early old age pensioners were more likely to be classified in the intermediate PF trajectory groups. Membership probabilities of PF trajectory groups for recipients of an individual retirement package and unemployment pension benefit were not significantly different from the probabilities of old-age retirees.

A previous study [9], which was conducted among older New Zealanders (*N* = 1368, 43.9% men, 39.9% professional occupation), identified PF trajectories, labelled as ‘recovering’, ‘maintaining’ and ‘declining’, and found retirement age not to be a significant predictor of membership across the PF trajectories. However, participants who reported to have been in paid work following retirement were more likely to belong to the ‘maintaining’ rather than the ‘recovering’ PF trajectory class. The average PF trajectory was generally on a higher level for individuals in non-professional occupations (declining vs. maintaining), low to medium socioeconomic status and among those with chronic conditions, a trend which was not found among participants characterized by good physical and economic well-being [9]. The present study extends our understanding of the factors related to post-retirement PF trajectory grouping among elderly retired workers. Among former executives and managers belonging to higher socioeconomic groups [11], older age at retirement was associated with a more favourable PF trajectory in old age, after adjustment for age, smoking, alcohol use, BMI, leisure time physical activity, cardiovascular disease, diabetes, and accounting for mortality risk over the 10-year period. The results are similar to the conclusion made by Szabo et al. [9] in that delaying retirement decisions in this group could benefit physical health in old age. Previous studies [19, 20] suggest an association with older age at retirement and lower all-cause mortality risk. Older age at retirement could infer better overall health and freedom from factors that limit work ability.

The PF trajectories identified by Jokela et al. [7], Mänty et al. [8] and Lahelma et al. [10] in higher socioeconomic groups have been observed to vary depending on the type of pension. The PF trajectories of women who continued to be occupationally active as municipal employees were most stable, followed by those of statutory and voluntary early retirees [10]. Another study found statutory and voluntary early retirement to be associated with better PF compared with being in the workforce [7]. Jokela et al. observed the PF of voluntary early pensioners in a high-SES group to resemble that of civil servants but to decline slightly faster after age 60 years [7]. Similarly, Mänty et al. observed the PF scores of professionals and managers decrease less among statutory pensioners, followed by part-time pensioners [8]. Our results were generally in line with these findings and found no evidence of benefits of early or part-time pension for post-retirement PF trajectory grouping in old age. In the present study, compared to old age pensioners, those receiving early old age pension benefits were more likely to be classified in the ‘intermediate and declining’ and ‘high and declining’ trajectories, relative to the ‘intact’ PF trajectory. These associations resemble those found for disability pensioners in the present study and hence suggest that early old age retirement may not be beneficial for physical functioning in old age in a non-manual labour high socioeconomic population.

In previous studies, the lowest PF scores have been observed among disability and ill-health pensioners [7, 8, 10]. However, the PF trajectories among these individuals were either stable and declined slowly [7], or improved substantially, in that those in lower occupational groups improved more than those in higher occupational groups [8]. The study by Mänty et al. found that irrespective of the type of pension the PF scores of individuals in the class of managers and professionals decreased after retirement more than in other occupational groups and increased the least among disability pensioners who had served as managers or professionals [8]. In the present study, were able to corroborate the findings of Jokela et al. (*N* = 7584, 69% men) in that disability pensioners were more frequent in the trajectory categories where PF level was low and remained low [7]. Compared to old age pensioners, those receiving disability pension benefits were at increased probability to be classified, in order of decreasing magnitude of the association, in the ‘consistently low’, ‘intermediate and declining’ and ‘high and declining’ trajectories, relative to the ‘intact’ PF trajectory, adjusting for age, smoking, alcohol consumption, BMI, LTPA, diabetes, cardiovascular disease, and mortality risk. This suggests that disability pensioners, regardless of various health factors, may be at risk of poorer PF. Mental health (53%) and musculoskeletal morbidity (18%) were major reasons for granting disability pensions in Finland in the year 2020 [21], highlighting potential risk of poorer PF attributable to pre-existing medical history and the importance of minimizing work-related physical and mental hazards.

We are not aware of previous studies which track the PF of unemployment pensioners and those receiving individual retirement packages. We found the trajectory group probabilities between old-age and unemployment retirees surprisingly similar. Although we observed no statistically significant differences, the point estimates were all smaller than one. Lack of significance is likely due low power as the unemployment retirement group was the smallest group in this sample. However, their prevalence in the category of intact PF was more than twofold higher than any other trajectory. This group of pensioners was characterized by younger age at retirement, younger age in the year 2000 and a lower prevalence of cardiovascular disease (data not shown). In the light of previous literature on the effects of unemployment the finding is surprising and runs against what we hypothesized. In a British cohort, a discontinuous work career was associated with both poorer physical and mental functioning at age 60 to 64 years [22]. Moreover, a prospective study found late-career unemployment to be associated with increased disability and mortality [23]. On the other hand, in the light of *activity theory* [5], unemployment and an earlier exit from the labour market could translate into increased time resources, which could then be allocated to activities that maintain physical health. Globally and across EU member states, Finland provides generous unemployment insurance net replacement rates [24], potentially alleviating economic difficulties following unemployment. While receipt of unemployment pensions necessitated prolonged unemployment preceding retirement, as managers and executives, participants in the study likely did not experience significant discontinuity during their working careers.

Individual retirement packages, a unique feature of the present study, were not associated with membership across the PF trajectories, comparing with old age pensioners. This would suggest that this group had relatively stable PF after retirement, which may resemble that observed for old age pensioners. Theory-wise, they unlikely represent a group of special interest regarding later PF and possible interventions targeted at maintaining PF should instead target vulnerable groups.

### Strengths and limitations

We studied retired businessmen who had first reported their retirement characteristics and then followed up for 10 years. Multiple assessments of physical functioning, performed using the validated RAND-36 Health Survey questionnaire [13], were modelled using LGMM analyses, which is a well-suited approach to describe longitudinal data and to detect change between and within unobserved groups. To control for some of the effects of sample attrition and healthy survivor effect we included simultaneous mortality risk adjustment during the 10-year observation period in our regression model. The men were characterized by high socioeconomic status and non-manual work history. Their positions or line of work were not known in detail, and as such no universal retirement age could be defined in this group consisting of managers and entrepreneurs. Retirement status was self-reported, and though this may be subject to recall bias, self-reported retirement age has been found to correlate positively with register information on the decrease in income from labour earnings [25]. Besides types of pension used by most studies, we were additionally able to distinguish between early old age pensions, part-time pensions, unemployment pensions, and receipt of an individual retirement package, a unique form of retirement rarely seen for occupational groups other than executives and managers. However, these types of pension benefit and their respective awarding criteria have changed with time and are subject to cohort effects. For example, while the lower age limits for old age and early old age pensions were very stable when the present cohort retired, that of unemployment pensions varied between 55 and 60 years. Besides these age criteria, working conditions and economic circumstances have also changed. Future studies focusing on more contemporary cohorts are therefore needed to address our findings.

We used data collection wave instead of age as time metric in constructing the PF trajectories. While we did not find evidence of significant classification uncertainty using this approach, differences in the participants’ age at baseline may affect their interpretation. To address this, we adjusted our analysis with age at baseline. Reverse causality, in that better PF would infer better work ability, enabling individuals to retire older and more frequently at the statutory retirement age, is possible. To control this, we adjusted the analyses with factors known to be associated with PF, including age, BMI, smoking, alcohol consumption, LTPA and key chronic diseases cardiovascular disease and diabetes. Despite this, we cannot exclude that the time between age at retirement and the measurements of their PF may have been long. We were not able to provide further information on the participants’ wealth or income, as the present study was originally set up to study cardiovascular disease. The advantage of having a study population homogenous in terms of sex and socioeconomic status is that the internal validity of the results can be high, although it can also limit the extent to which they are generalizable to other occupational or socioeconomic groups.

## Conclusions

In conclusion, we found no evidence to support potential harmful effects of later retirement on physical health among retired male executives and managers, as we did not observe a significant difference between early retirement vs. old age pension in association with PF trajectories group membership. Relative to old age pensioners, disability and early old age pensioners were disadvantaged in terms of PF trajectories in old age, a trend that was not observed for unemployment pensioners and those receiving individual retirement packages, who better maintained their PF. The results suggest potential benefits to PF trajectories in old age among executives and managers who retired.

## Supplementary Information

**Additional file 1: Supplementary Table 1.** Model Fit Statistics, Group Sizes and Average Latent Class Probabilities for Most Likely Class Membership. Figures. Reproduced with permission from [15]. **Supplementary Fig. 1.** Individual observations belonging to each of the five identified physical functioning trajectories. Reproduced with permission from [15].

## Data Availability

Inquiries regarding the datasets used and/or analysed during the current study can be directed to the principal investigator (Timo E Strandberg) of the Helsinki Businessmen Study.

## Abbreviations

PF: Physical functioning
mOR: Multinomial odds ratio
HBS: Helsinki Businessmen Study
LGMM: Latent growth mixture modelling
LTPA: Leisure time physical activity
SD: Standard deviation
BMI: Body mass index
