# Supplementary material for: Retirement as a predictor of physical functioning trajectories among older businessmen
Source: BMC Geriatr. 2022 Apr 4;22:279. doi: 10.1186/s12877-022-03001-x (PMC8981673; doi:10.1186/s12877-022-03001-x)

Supplementary Material Legend

Supplementary Table 1. Model Fit Statistics, Group Sizes and Average Latent Class Probabilities for Most Likely Class Membership

Figures. Reproduced with permission from (15).

Supplementary Figure 1. Individual observations belonging to each of the five identified physical functioning trajectories. Reproduced with permission from (15).

Supplementary Table S1. Model Fit Statistics, Group Sizes and Average Latent Class Probabilities for Most Likely Class Membership.

				Information criteria				Group size (Average latent class probability for most likely latent class membership)					
**Classes**	**LL**	**Scaling**	**Free parameters**	**AIC**	**BIC**	**aBIC**	**Entropy**	n_1	n_2	n_3	n_4	n_5	n_6
1	-21939	1.88	13	43904	43977	43935	1.000	1991 (1.00)					
2	-20815	1.26	27	41684	41835	41749	0.742	960 (0.95)	1031 (0.91)				
3[b]	-20597	1.49	38	41270	41483	41362	0.712	742 (0.93)	818 (0.84)	431 (0.81)			
4[c]	-20495	1.31	49	41087	41361	41206	0.691	610 (0.78)	198 (0.80)	517 (0.92)	666 (0.78)		
**5[d]**	**-20453**	**1.24**	**54**	**41009**	**41312**	**41140**	**0.712**	**556 (0.78)**	**191 (0.79)**	**461 (0.84)**	**631 (0.78)**	**152 (0.88)**	
6[e]	-20422	1.18	65	40973	41337	41131	0.658	535 (0.73)	189 (0.78)	139 (0.66)	619 (0.79)	377 (0.66)	132 (0.79)

Note. LL = loglikelihood, scaling = Robust maximum likelihood scaling factor, AIC = Akaike information criterion, BIC = Bayesian information criterion, aBIC = sample size adjusted Bayesian information criterion.

[b]Parameter restrictions in class 3: var(Q)=0, cov(I,Q)=0, cov(S,Q)=0.

[c]Parameter restrictions in class 2: var(Q)=0, cov(I,Q)=0, cov(S,Q)=0, and in class 3: var(Q)=0, cov(I,Q)=0, cov(S,Q)=0.

[d]Parameter restrictions in class 2: var(Q)=0, cov(I,Q)=0, cov(S,Q)=0, and in class 3: var(I) = 0, var(S), var(Q)=0, cov(I,Q)=0, cov(I,S) = 0, cov(S,Q)=0, and in class 4: intercept(Q)=0, var(I) = 0, var(Q)=0, cov(I,Q)=0, cov(I,S) = 0, cov(S,Q)=0.

[e]Parameter restrictions in class

Supplementary Figure S1

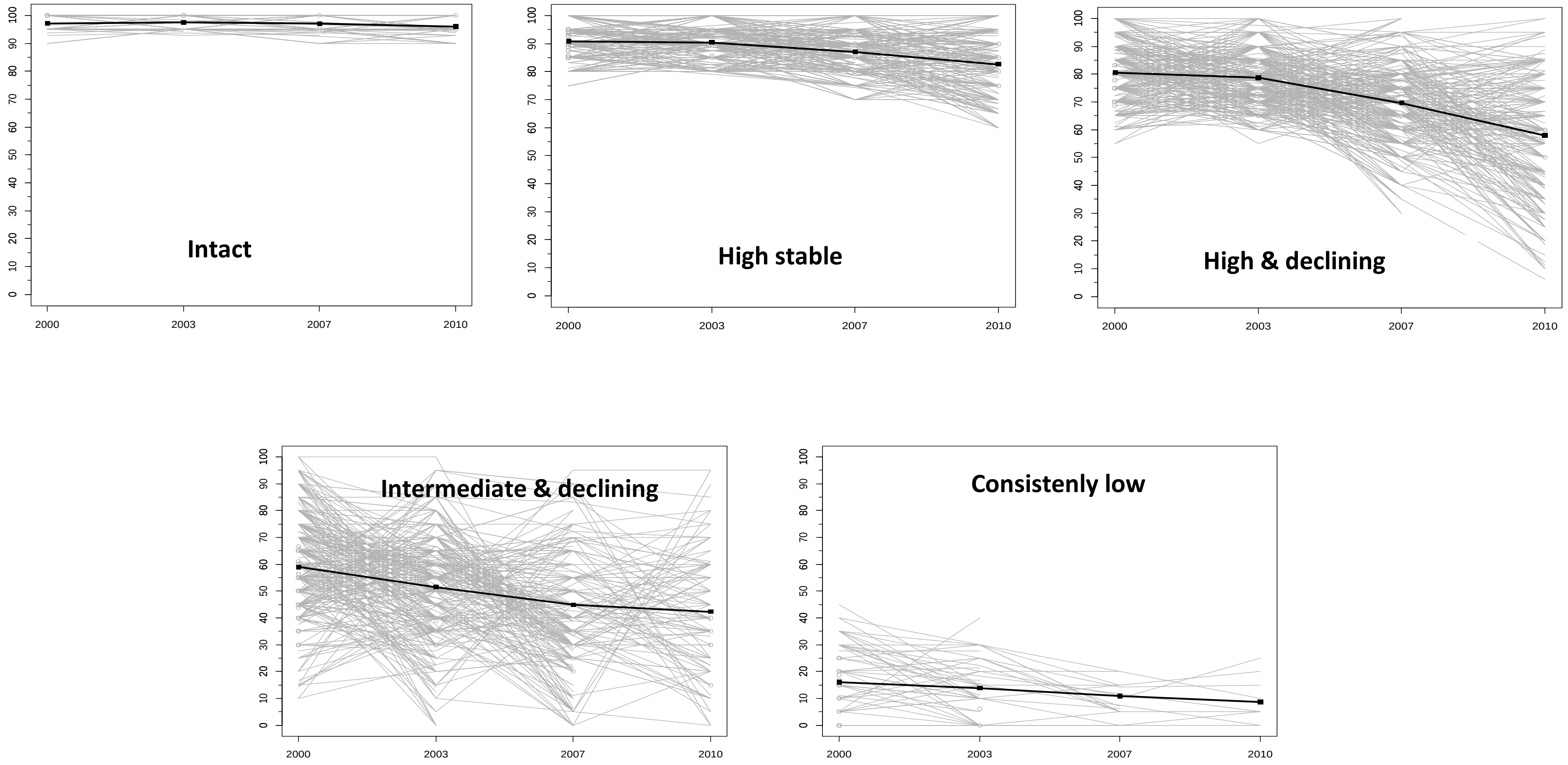

Supplement: Supplementary file 1 — Additional file 1: Supplementary Table 1. Model Fit Statistics, Group Sizes and Average Latent Class Probabilities for Most Likely Class Membership. Figures. Reproduced with permission from [15]. Supplementary Fig. 1. Individual observations belonging to each of the five identified physical functioning trajectories. Reproduced with permission from [15]. [file 12877_2022_3001_MOESM1_ESM.pdf]
